# Bibliography

**Published:** 1903-01

**Authors:** 


### Bibliography.

Anatomy and Histology of the Mouth and Teeth. By I. Norman Broomell, D.D.S., Professor of Dental Anatomy, Dental Histology, and Prosthetic Technics in the Pennsylvania College of Dental Surgery, Philadelphia. Second edition, revised and enlarged, with three hundred and thirty-seven illustrations. Blakiston's Son & Co., Philadelphia, 1902.

This work of Dr. Broomell was very fully reviewed when the first edition was published, and it must be gratifying to the author that the good opinion then entertained for this original work has been fully sustained by the dental reading public. Scientific works do not, as a rule, sell rapidly.

The author, "after due consideration," concluded to retain the ninety-three pages devoted to general anatomy of the mouth. This was criticised in the review of the first edition, and it is still the opinion of the writer that its retention is a mistake, for, however well prepared, it adds nothing to the character of the book, but does help to give it bulk, a serious evil in the production of a text-book. All students will get their anatomy from books devoted to that specialty.

The author says, "In the description of the teeth the terms 'superior' and 'inferior' have been changed to 'upper' and 'lower,' and the term 'palatial,' as applied to one of the teeth surfaces, has been discarded and the word 'lingual' substituted."

A chapter dealing with "Embryology of the Mouth" has been added, and also one on "Anomalies of Tooth Form and Structure;" also fifty-three new illustrations have been added, all of which, with one or two exceptions, are the original work of the author. This constitutes the especial merit of the book. Whatever exceptions may be taken to some of the author's conclusions, the book stands without an equal in dental literature. With the exception noted, that of anatomy, this work is the result of earnest, painstaking effort to reach truth by personal investigation, and then to give the results, not from drawings, in which the personal equation can never be eliminated, but from photographs and microphotographs, in the production of which the author has few if any superiors. No dental book with which the reviewer is familiar has ever attempted the same character of work, and it therefore stands not only unique in its originality, but in many respects the one work which may be referred to as a finality.

The new chapter on Embryology is a valuable contribution. While it may not contain much that is new, it does make the subject much clearer through the very beautiful illustrations that accompany the text.

The chapter on "Anomalies" is not quite so satisfactory to the reviewer. It is not to be supposed that the author has not given study to these conditions, yet this would seem to be indicated by his sub-chapter on "Supernumerary Teeth." He says, "All teeth appearing in the mouth in addition to the normal number are designated as supernumerary teeth. While the author has, of course, the right to classify, as he deems best, yet his definition is at variance with accepted views. Supernumerary teeth have a distinct typical character, which the writer described in 1863 (see *Dental Times*), and it is impossible properly to class these as the author has done. They have no resemblance either in form or general character to the supernumerary. Sir John Tomes called these in 1849 "supplemental," recognizing their normal character. The not uncommon occurrence of four lateral incisors, all perfect teeth, three perfect premolars, etc., should have been noted. It is true, the author as quoted writes of teeth "in addition to the normal number," but this is a very imperfect way of describing these extra developments, especially where students are involved. The writer has known of instances where one of these extra incisor teeth has been extracted through ignorance of the important fact that they are normal to that special jaw. It is to be hoped that when a third edition is called for this chapter will be rewritten, as it is not quite in character with other portions of the book.

THE CARE OF THE TEETH. By Samuel A. Hopkins, M.D., D.D.S., Professor of Theory and Practice of Dentistry in Tufts College Dental School. D. Appleton & Co., New York, 1902.

This valuable book of one hundred and fifty pages opens with this basic truth in the "Introduction," that "Observations made during an active practice of over twenty years have convinced the writer of the truth of two propositions,—first, that a large proportion of dental operations are preventable, and second, that a large proportion of the world's inhabitants are ignorant of how to prevent them." The author, with this very correct view in his mind, has written this little book in the hope that it may do something towards enlightening the minds of those who may be fortunate enough to read and absorb the contents of its pages.

In the first chapter on the "History of Dentistry. Uses of Teeth," the following statement is made, that seems hardly warranted by the experience of at least one of his readers: "College statistics show that the record of athletes in their studies is fully up to the general average of college work." While it is true that college men frequently point with pride to some exceptional man as proof of this assertion, it does not, as a rule, accord with the reviewer's experience. The amount of time spent in athletic work in colleges is certainly a serious loss to the individual. Some can make it up, others cannot.

Chapter III., on "Predisposing Causes. Lack of Exercise," is a very valuable and instructive essay upon the subject of proper mastication. There can be no question that the foods as prepared under modern civilization have had much to do with caries of the teeth, but is it true that mastication, or rather lack of mastication, is the prominent factor? Is it not true, even with our delicately prepared foods, that teeth become more dense as age advances? This, of course, does not militate against the idea that use increases quality of tooth-structure, but it does against that somewhat prevalent notion that prepared food will eventually produce an edentulous race.

In the chapter on "Food" there is this statement, that seems to require qualification: "We know that grain grown upon lands rich in calcium salts is of vastly greater nutritive value than that grown upon poor and exhausted lands. . . . Observations made of the teeth of a large number of school children in Sweden show that in the lime regions, where the grains contain a high percentage of calcium salts, the teeth are very much stronger," etc.

This may be true, but the author would have strengthened his statement by giving his authority. The experience of the reviewer in some extended practice in a region with a high percentage of calcium salts does not bear out his assertion; indeed, teeth seem to have been, by this experience, very little affected by the peculiar conditions of the soil. It is a serious question whether those who so strenuously advocate "whole wheat food" are not forgetting a well-known fact that the average individual has more of lime in his system than is necessary for the nutrition of the hard tissues, and that to increase it may result in depositions of calculi in various organs, as well as masses upon the teeth. While it is true that with young persons a diet largely composed of cereals seems to increase density of tooth-structure, we yet lack laboratory experimentation to test this supposed fact of clinical experience.

"Bread," our author says, "should never be eaten until it is at least twenty-four hours old." This may be satisfying to our author, but it is feared his advice, however good, will pass unheeded, especially with those in our large cities who must depend on the baker. Home-made bread, when properly prepared, is satisfying twenty-four or more hours old, but the making of bread in the average household is almost a lost art. This does not invalidate our author's argument; in fact, it rather enforces it.

On page 90 the author, after giving a very satisfactory explanation of the trouble occasioned by the development of the second molar of the lower jaw, gives advice which to the reviewer seems uncalled for if his clinical observations are of any value. He has never met with a second molar in the lower jaw that could not be relieved by deep lancing. The reflex disturbance from this tooth is the result of pressure upon the inferior dental nerve and the reaction of this pressure upon the pulp in the passage of the tooth from the ramus to assume the direct vertical and normal position. The advice, therefore, in desperate cases to extract the first molar is not only uncalled for, but will result in a maze of future difficulty hardly possible to overcome.

The chapter on "Prevention of Caries or Decay" is the most valuable in the book. People must be made to understand that the only effectual cleaning of the teeth must be through the care given by the dentist at regular stated intervals, and the only way to effect this much-needed reform is to educate the dentist up to an appreciation of this fact. He has not yet reached this desirable stage of mental progress. This work our author has attempted to do. We could wish that this book could be placed in the hands of every young mother for the good this chapter alone might accomplish.

Every dentist must agree with the author, in the chapter on

"Toothache and the Teeth of the Poor," that it is a crying necessity that the poor should receive attention, but while this is true, there seems no possibility of this being effectually carried out, unless some of our millionaires will endow institutions for the purpose. It will never be possible for individual dentists to do this work, even if willing to give a day each week to this helpful service. The peculiarities of dental practice will act as a perpetual bar to this, and it seems useless to urge it. The work of the colleges in their dispensaries is probably the nearest approach to relief that we can hope to have. In some of our large cities this help has reached enormous proportions. The colleges do not all charge for the service. In the institution with which the reviewer is connected no charge is made for work in the operative branch, except that for gold operations. It is a question whether a small charge would not avoid the danger of pauperizing in this direction.

More space has been given to this little book than its size seems to warrant, but bulk is not always an indication of quality, and this is particularly true of Dr. Hopkins's work.

It is worthy to occupy a place in every family, and there it legitimately belongs. Whether we agree with it all or not, the tendency is towards a higher standard of health through more perfect sanitary conditions in the oral cavity; and that seems to the reviewer to be the most important thing given to the dentist for his work in the twentieth century.

